# The structural relationship of job stress, job satisfaction, organizational commitment, and turnover intention among youth sports education leaders in Korea

**DOI:** 10.3389/fpsyg.2024.1385993

**Published:** 2024-11-18

**Authors:** Myung Kyu Jung, Tae Gyeom Jung, Min Woo Jeon, Ji Hae Lee

**Affiliations:** ^1^Department of Physical Education, Kyung Hee University, Yongin-si, Gyeonggi-do, Republic of Korea; ^2^Department of Physical Education, Shinhan University, Uijeongbu-si, Gyeonggi-do, Republic of Korea

**Keywords:** Korea youth sports education leaders, job stress, job satisfaction, organizational commitment, turnover intention

## Abstract

**Introduction:**

This paper is a study aimed at providing insights for developing effective human resource management strategies suitable for Korean youth sports education facilities, by elucidating the multifaceted relationships among job stress, job satisfaction, organizational commitment, and turnover intention. Through the research, it was academically suggested that the relationships among job stress, job satisfaction, organizational commitment, and turnover intention of Korean youth sports education facility workers could be structurally and complementarily manifested. To fulfill this objective, data were gathered using snowball sampling from Sports leaders who have experience or are engaged in sports education facilities for youth in Korea as of 23 years. A total of 384 responses were analyzed using frequency, technical statistics, and confirmatory factor, reliability, correlation, and structural equation model analyses.

**Results:**

Through these analyses, this study found that job Stress, job satisfaction, organizational commitment were significant as both direct and indirect influences on turnover Intention. Intervention strategies should focus on fostering the positive emotions that stem from Intermediation, mitigation, and improvement of job stress.

## 1 Introduction

In Korea, securing excellent human resources through human resource management in the operation of sports facilities is a critical factor that determines the success or failure of management. Particularly in the case of the sports facility industry in Korea, which is centered around educational services, the importance of securing excellent human resources is increasingly emphasized due to the nature of the industry, where the interaction between service providers and consumers is crucial. Moreover, the loss of competent human resources can have a fatal impact on organizational competitiveness (Morrell et al., 2004; Seo, 2018). Therefore, it is necessary to develop effective human resource management strategies to prevent the departure and turnover of affiliated sports instructors.

However, in the case of youth sports facilities centered on educational services in Korea, the reality is that the level of departure and turnover of sports instructors is further exacerbated due to exposure to various job environments such as vehicle operation, counseling, and promotion, in addition to educational guidance (Choi and Jung, 2020; Kang and Jung, 2023; Kim and Jung, 2021; Jung et al., 2023; Lee, 2021; Yoon et al., 2019). Particularly, as can be inferred from reports like “Childcare Teachers Trembling from Fear of Power Abuse by ‘Mom Cafes’ and Parents” (Lee, 2020) and “‘Taekwondo Studios as Childcare Centers’… Reality Reflected in Sympathetic SNS Posts” (Cho, 2019), youth sports education facilities in Korea are not only focused on education but also emphasize a childcare role. This unique environmental characteristic leads to an increased level of negative emotions experienced in the job due to the selfishness of parents who only care about their own children, often represented by power abuse. Consequently, the difficulty of securing competent human resources in youth education-centered sports facilities operating in Korea is becoming increasingly severe that they are called “picking stars in the sky” (Lee, 2021).

In light of these issues, this study aims to provide insights into effective human resource management strategies to prevent the departure and turnover of employees. It focuses on turnover intention, which is the most critical predictive factor in forecasting the departure and turnover of employees in an organization (Bluedorn, 1982; Brown and Peterson, 1993; Igbaria and Siegel, 1992; Rajan, 2015; Yang et al., 2014). The study seeks to elucidate the multidimensional impact among key psychological factors related to human resource management strategies, namely job stress, job satisfaction, and organizational commitment.

According to previous studies on the variables mentioned, job satisfaction, organizational commitment, job stress, and turnover intention are structurally related in various aspects. Specifically, in situations where job stress, job satisfaction, and organizational commitment act as predictor variables for turnover intention (Arshadi and Damiri, 2013; Chen and Kao, 2011; Cotton and Tuttle, 1986; Dodanwala et al., 2023; Elci et al., 2012; Fasbender et al., 2019; Iqbal et al., 2014; Jehanzeb et al., 2013; Jou et al., 2013; Kuo et al., 2012; Maertz and Campion, 2004; Romeo et al., 2020; Williams and Hazer, 1986; Yan et al., 2021a, 2021b), job stress has a significant predictive relationship with job satisfaction and organizational commitment (Aghdasi et al., 2011; Arshadi and Damiri, 2013; Chen and Kao, 2011; Jepson and Forrest, 2006; Kuo et al., 2012; Trivellas et al., 2013; Wang et al., 2020; Wu et al., 2021). In this case, job satisfaction also plays a significant role in enhancing organizational commitment (Iqbal et al., 2014; Mowday et al., 1982; Wang et al., 2020; Williams and Hazer, 1986). In other words, job stress, job satisfaction, and organizational commitment form a structural relationship and play a complementary role in mediating an individual’s intention to leave.

However, further discussion is required in exploring human resource management strategies useful for youth sports education facilities by clarifying the relationships between job stress, job satisfaction, organizational commitment, and turnover intention. This need arises despite the differences in individual psychological factors and their relationships due to job characteristics and employment types, as pointed out in related studies conducted with organizational members in South Korea (Heo, 2001; Kim and Park, 2019; Kim and Jung, 2021; Kim et al., 2012; Kim and Yu, 2008; Kwon et al., 2013; So et al., 2009; Jang and Suh, 2020; Lee and Jeon, 2021). Specifically, there has been a lack of direct research on sports instructors working in youth sports facilities. Moreover, the studies conducted in Korea have primarily focused on the fragmentary interpersonal relationships between variables like job stress, job satisfaction, organizational commitment, and turnover intention. Discussions on the results have been concentrated on applying inferences drawn from different subjects to employees at youth sports facilities. This limitation highlights the indirect need for studies on the structural relationships between these variables.

Based on the above content, this study aims to elucidate the structural relationship among job stress, job satisfaction, organizational commitment, and turnover intention of employees working in youth sports facilities. As described, given the increasing need for effective human resource management strategies to prevent staff turnover and attrition in these facilities, this study focuses on turnover intention, a major predictive variable of actual turnover among organizational members (Aryee et al., 2002; Bluedorn, 1982; Brown and Peterson, 1993; Igbaria and Siegel, 1992; Rajan, 2015; Yang et al., 2014). It explores the structural relationships of key psychological factors in human resource management strategies, namely job stress, job satisfaction, and organizational commitment, in various aspects. This research is expected to provide academically significant insights and practically effective information for developing psychological coping strategies, particularly tailored to the characteristics of Korean youth sports education facilities.

Based on the described content, this study intends to use Structural Equation Modeling (SEM) to reveal the structural relationships of job stress, job satisfaction, organizational commitment, and turnover intention among sports instructors working in youth-centered sports education facilities. Through this, the study aims to provide various implications for effective human resource management strategies that are suitable for securing excellent human resources, specifically tailored to the characteristics of Korean youth sports education facilities.

## 2 Hypotheses development

Turnover intention generally refers to an individual’s behavioral intention to leave their job, meaning the intention to leave an organization or profession (Cho et al., 2022; Rajan, 2015). Moreover, in understanding and predicting an organizational member’s departure, turnover intention can be utilized as a predictive factor (Aryee et al., 2002; Bluedorn, 1982; Brown and Peterson, 1993; Cho et al., 2022; Igbaria and Siegel, 1992; Rajan, 2015; Yang et al., 2014), and hence has been approached as a key element in human resource management strategies.

In this context, formulating human resource management strategies to mediate the turnover intention of organizational members, specifically sports instructors, is of great significance for Korean youth sports education facilities, where securing excellent human resources is becoming increasingly challenging. Moreover, in mediating turnover intention, the development of effective human resource management strategies begins with an understanding of the influence of psychological factors related to an individual’s intention to leave.

Job satisfaction and organizational commitment are among the typical predictive variables for turnover intention. The job satisfaction and organizational commitment referred to here are psychological concepts, respectively indicating the positive emotions gained through experiences related to the job and the sense of belonging felt in the organization (Meyer et al., 1993; Choi and Jung, 2020; Locke, 1976; Williams and Anderson, 1991; Yang and Lee, 2018). As pointed out by numerous researchers (Dodanwala et al., 2023; Iqbal et al., 2014; Oh et al., 2007; Porter and Steers, 1973; Romeo et al., 2020; Tett and Meyer, 1993; Yan et al., 2021a, 2021b), experiencing positive emotions toward one’s job and organization can lead to various psychological benefits within the organization, such as mediating turnover intention. For instance, Jeon et al. (2023) indicated that job satisfaction and organizational commitment could mediate the turnover intention of Korean martial arts facility workers. Furthermore, several studies focusing on employees in various Korean sports facilities (Chon, 2007; Chung and Kang, 2014; Kim et al., 2012; Seo, 2018; Seo, 2012) have demonstrated the significant role of job satisfaction and organizational commitment in turnover intention.

Conversely, as argued in previous studies, job stress can act as an effective psychological factor in mediating job satisfaction, organizational commitment, and turnover intention. Job stress refers to the negative psychological and physiological responses of an individual caused by various stress factors related to job situations, and is a type of stress associated with work conditions (Beehr and Newman, 1978; Jeong et al., 2018; Judge and Colquitt, 2004). Indeed, numerous past studies have explained that job stress can determine the levels of job satisfaction, organizational commitment, and turnover intention (Aghdasi et al., 2011; Arshadi and Damiri, 2013; Beehr and Bhagat, 1985; Bhatti et al., 2016; Chen and Kao, 2011; Elci et al., 2012; Gupta and Beehr, 1979; Jepson and Forrest, 2006; Jou et al., 2013; Kuo et al., 2012; Novitasari, 2020; Pines and Keinan, 2005; Trivellas et al., 2013; Wang et al., 2020; Wu et al., 2021). For example, Jeong and Jung (2024), Jung and Park (2024), and Choi et al. (2013) suggest the effectiveness of job satisfaction, organizational commitment, and turnover intention presented through related research on Korean youth sports facilities.

In this context, considering that job satisfaction can lead to organizational commitment (Iqbal et al., 2014; Mowday et al., 1982; Oh et al., 2007; Wang et al., 2020; Williams and Hazer, 1986), it is believed that job stress, job satisfaction, and organizational commitment will act as effective psychological factors from the perspective of mediating turnover intention. In other words, job stress, job satisfaction, and organizational commitment can mediate the turnover intention of youth sports education instructors through their structural and mutually complementary effects.

However, despite the possibility of multidimensional manifestations in the validity of the relationships among psychological factors of sports instructors due to job characteristics and employment types, as previously described (Cohen and Hudecek, 1993; Heo, 2001; Jang and Suh, 2020; Kim and Jung, 2021; Kim and Park, 2019; Kim et al., 2012; Kim and Yu, 2008; Kwon et al., 2013; Lee and Jeon, 2021; So et al., 2009), there is a lack of direct research on the mentioned aspects concerning youth sports education instructors. Additionally, previous studies conducted in Korea on job stress, job satisfaction, organizational commitment, and turnover intention have not seriously considered the structural causality among these variables. Consequently, the results are often limited to applying findings from other professions to youth sports education instructors, which restricts understanding the relationships between job stress, job satisfaction, organizational commitment, and turnover intention of youth sports education instructors in Korea in terms of structural causality.

In light of this content, to develop useful human resource management strategies suitable for Korean youth sports education facilities, it is necessary to elucidate the empirical structural causal relationships among job stress, job satisfaction, organizational commitment, and turnover intention. Therefore, this study sets the following research hypotheses to clarify the multifaceted relationships among job stress, job satisfaction, organizational commitment, and turnover intention of youth sports education instructors.

*Hypothesis 1*: Job stress among Korean youth sports education instructors will have a significant impact on job satisfaction.

*Hypothesis 2*: Job stress among Korean youth sports education instructors will have a significant impact on organizational commitment.

*Hypothesis 3*: Job stress among Korean youth sports education instructors will have a significant impact on turnover intention.

*Hypothesis 4*: Job satisfaction among Korean youth sports education instructors will have a significant impact on organizational commitment.

*Hypothesis 5*: Job satisfaction among Korean youth sports education instructors will have a significant impact on turnover intention.

*Hypothesis 6*: Organizational commitment among Korean youth sports education instructors will have a significant impact on turnover intention.

## 3 Methodology

### 3.1 Sample and procedure

This study aimed to achieve its research objectives by collecting data over a period of approximately 2 months, from December 2023 to January 2024, using snowball sampling, a non-probability sampling method. Data was initially collected from a small number of Korean youth sports leaders. Later, an online questionnaire was provided to other Korean youth sports leaders, including the organizations to which the participants belonged. The participants were physical education instructors in South Korea who have worked or are currently working in youth sports education facilities as of 2023. They responded to the survey based on self-evaluation after receiving an online survey link through social media (PO1-202312-01-029).

Considering the limitations of online surveys, we added items like “Please respond with <7, Strongly Agree>” to enhance the reliability of the responses. We then went through a data cleaning process, which involved filtering out responses that differed from the requested ones in certain items or showed specific patterns (e.g., 111,111 or 777,777). Ultimately, 384 individuals were selected for the study. The general characteristics of the study subjects were as follows: Firstly, in terms of gender, there were 257 males (66.9%) and 127 females (33.1%). Secondly, by age group, there were 177 individuals in their 20s (46.1%) and 207 individuals in their 30s (53.9%).

### 3.2 Measurement

The questionnaire was comprised of four content areas: Job Stress, Job Satisfaction, Organizational Commitment, Turnover Intention. Existing scales were adapted to fit the context. Firstly, the job stress items were reconstructed as a single factor (5 items) by modifying and supplementing the questions used in the studies of Hong and Lee (2000), Jung et al. (2023) and Kang and Jung (2023), targeting Korean sports instructors. Next, the job satisfaction items were reconstituted as a single factor (4 items) by revising and enhancing the questions used in the research of Williams and Anderson (1991), and Jeon et al. (2023). Following that, the organizational commitment items were also restructured into a single factor (4 items) by adapting and refining the questions from the studies of Mowday et al. (1979) and Jeon et al. (2023). Lastly, the turnover intention was reconstituted as a single factor (4 items) based on, and modified from, the items used in the research of Bluedorn (1982) and Mowday et al. (1982).

Each of the items used was validated for content validity by a total of five sports science PhDs with experience working in Korean youth sports education facilities. Apart from the demographic characteristics of the research subjects (4 items), each item was measured using a Likert 7-point scale.

### 3.3 Data analysis

In this study, to achieve the research objectives, data collected through self-administered responses were analyzed using SPSS v28.0 and AMOS v21.0. The analysis was conducted as follows: First, frequency analysis was used to understand the demographic characteristics of the study participants. Second, the validity and reliability of the measurement tools were assessed through Confirmatory Factor Analysis (CFA) model validation and reliability analysis based on Cronbach’s *α*. Third, correlation analysis was performed on the unidimensionally established job stress, job satisfaction, organizational commitment, and turnover intention to address multicollinearity issues and validate discriminant validity. Fourth, Structural Equation Modeling (SEM) analysis was conducted to test the set research hypotheses. Fifth, based on the characteristics of the research model set for hypothesis testing, the significance of specific indirect effects was examined through estimation based on phantom variables and bootstrapping to assess the significance of specific indirect effects.

## 4 Results

Based on SEM-related characteristics, the univariate normality of the data was reviewed prior to examining the structural relationship between job stress, job satisfaction, organizational commitment, and turnover intention (see Table 1). West et al. (1995) suggested that data normality can be assessed using skewness (test criterion ±2) and kurtosis (test criterion ±7). In relation to this, Bae (2017) advised that the kurtosis value detected in SPSS should be noted as already subtracted by 3, and based on this, each value was verified. The results showed skewness ranging from −0.638 to 0.953 and kurtosis from 2.428 to 3.200, suggesting that normality was satisfied.

**Table 1:** Means, standard deviations, and bivariate correlations.

	JST	JSA	OC	TI
Job stress (JST)	1			
Job satisfaction (JSA)	−0.703**	1		
Organizational commitment (OC)	−0.689**	0.792**	1	
Turnover intention (TI)	0.728**	−0.719**	−0.708**	1
Mean	3.074	4.837	4.553	3.216
Standard deviation	1.385	1.608	1.580	1.468
Skewness	0.953	−0.638	−0.562	0.807
Kurtosis	3.200	2.557	2.428	2.715

**p < 0.01.

### 4.1 Measurement model

To examine the validity and reliability of the measurement tool, a Confirmatory Factor Analysis (CFA) model based on measurement model analysis and reliability analysis based on Cronbach’s *α* coefficient were conducted (see Table 2). As a detailed explanation of the analysis results, first, results satisfying the model fit indices proposed by the American Psychological Association (APA) were detected (*χ*^2^ = 937.872, df = 203, CFI = 0.951, TLI = 0.944, SRMR = 0.0397, RMSEA = 0.097). Subsequent additional analysis detected measures of AVE above 0.5 (0.678 ~ 0.823), CR above 0.7 (0.927 ~ 0.949), and α above 0.7 (0.973 ~ 0.981), and based on these, it was inferred that convergent validity and reliability were satisfied (Bae, 2017; Garrido et al., 2016; Schermelleh-Engel et al., 2003; Steiger, 1990). Moreover, it was confirmed that the lowest AVE index (0.678) was higher than the square of the maximum correlation coefficient (0.792) (0.627), and based on this, it was inferred that discriminant validity was also satisfied (Anderson and Gerbing, 1988; Fornell and Larcker, 1981).

**Table 2:** Measurement properties of the first-order latent constructs.

Construct and item	*λ*	*t*	*α*	AVE	C.R.
Job stress (JST)			0.975	0.749	0.947
JST1 (Level of fatigue due to job duties)	0.965				
JST2 (Experience of headaches due to job duties)	0.976	55.257			
JST3 (Experience of indigestion due to job duties)	0.985	59.719			
JST4 (Level of anger due to thoughts about work)	0.976	55.225			
JST5 (Level of anxiety regarding job performance)	0.834	27.215			
JST6 (Level of worry and concern about work)	0.830	26.852			
Job satisfaction (JSA)			0.981	0.726	0.941
JSA 1 (Satisfaction with current job)	0.986				
JSA 2 (Level of creativity expressed in job performance)	0.941	49.158			
JSA 3 (Level of satisfaction with superiors)	0.892	36.451			
JSA 4 (Level of satisfaction with supervisory functions of superiors)	0.912	40.504			
JSA 5 (Level of satisfaction with colleagues)	0.907	39.451			
JSA 6 (Sense of responsibility for work among colleagues)	0.991	88.349			
Organizational commitment (OC)			0.973	0.678	0.927
OC 1 (Level of identification with organizational issues)	0.906				
OC 2 (Satisfaction through comparison with other organizations)	0.940	32.482			
OC 3 (Level of hope for development and growth of the affiliated organization)	0.953	33.941			
OC 4 (Pride as a member of the affiliated organization)	0.922	30.608			
OC 5 (Level of Value assessment for organizational tasks)	0.929	31.358			
OC 6 (Evaluation of individual job contribution to organizational growth)	0.904	28.914			
Turnover intention (TI)			0.977	0.823	0.949
TI 1 (Experience of desiring to leave the organization)	0.951				
TI 2 (Establishing plans and gathering information to leave the organization)	0.960	43.901			
TI 3 (a desire to move to a similar industry)	0.956	42.966			
TI 4 (the desired level of turnover to other industries)	0.958	43.500			

Model fit: *x*^2^ = 937.872, df = 203, comparative fit index (CFI) = 0.951, Tucker–Lewis index (TLI) = 0.944, standardized root mean square residual (SRMR) = 0.0397, root mean square error of approximation (RMSEA) = 0.097. λ = Factor loading; α = Cronbach’s alpha; CR = Composite reliability; AVE = Average variance extracted.

### 4.2 Hypotheses testing

Prior to hypothesis testing (see Table 3), the structural equation model fit was checked, and it was found that the results exceeded the verification criteria of the indices suggested by APA (*χ*^2^ = 937.872, df = 203, CFI = 0.951, TLI = 0.944, SRMR = 0.0397, RMSEA = 0.097). Based on these results, it was inferred that the structural equation model set up in this study, that is, the research model for hypothesis testing, met the required fit criteria (Bae, 2017; Garrido et al., 2016; Schermelleh-Engel et al., 2003; Steiger, 1990).

**Table 3:** Results of structural equation modeling.

Path of Latent Variables	Direct effect	Indirect effect	*p*-value	Hypothesis testing
JST → JSA	−0.801(−0.682)***		<0.001	Supported
JST → OC	−0.310(−0.273)***		<0.001	Supported
JSA → OC	0.582(0.602)***		<0.001	Supported
JSA → TI	−0.199(−0.227)***		<0.001	Supported
JST → TI	0.383(0.372)***		<0.001	Supported
OC → TI	−0.267(−0.295)***		<0.001	Supported
JST → JSA → TI		0.159**	<0.01	Supported
JST → OC → TI		0.083**	<0.01	Supported
JST → JSA → OC → TI		0.125**	<0.01	Supported

Model fit: *x*^2^ = 937.872, df = 203, comparative fit index (CFI) = 0.951, Tucker–Lewis index (TLI) = 0.944, standardized root mean square residual (SRMR) = 0.0397, root mean square error of approximation (RMSEA) = 0.097; ***p < 0.001, **p < 0.01. JST, job stress; JSA, job satisfaction; OC, organizational commitment; TI, turnover intention.

**Figure 1:**
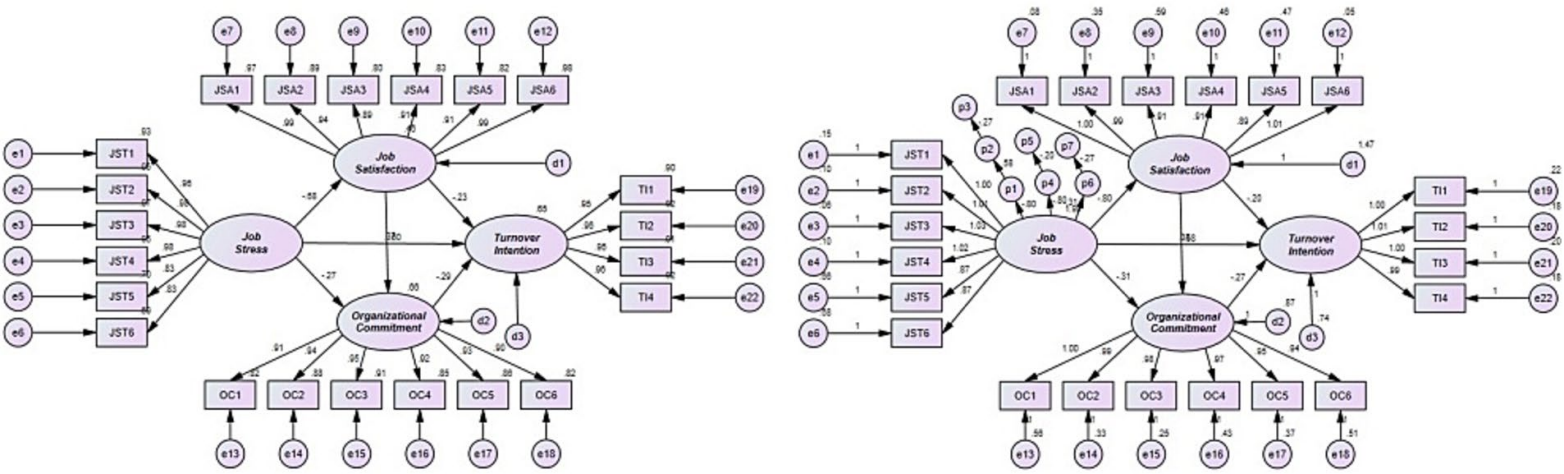
Results of the structural equation model analysis (left) and Phantom variables structural equation model analysis (right).

Based on the estimated model fit, the results of testing the research hypotheses set to clarify the structural relationships between job stress, job satisfaction, organizational commitment, and turnover intention among youth sports instructors are as follows (see Table 3 and Figure 1). Firstly, the statistical significance of the impact of job stress on job satisfaction, organizational commitment, and turnover intention was verified, leading to the acceptance of h1 (*b* = −0.801, *β* = −0.682, *p* < 0.001), h2 (*b* = −0.310, *β* = −0.273, *p* < 0.001), and h3 (*b* = 0.383, *β* = 0.372, *p* < 0.001). Next, the statistical significance of the impact of job satisfaction on organizational commitment and turnover intention was verified, leading to the acceptance of h4 (*b* = 0.582, *β* = 0.602, *p* < 0.001) and h5 (*b* = −0.199, *β* = −0.227, *p* < 0.001). Lastly, the statistical significance of the impact of organizational commitment on turnover intention was verified, resulting in the acceptance of h6 (*b* = −0.267, *β* = −0.295, *p* < 0.001).

On the other hand, Bae (2017) argued that even if indirect effects are not set as research hypotheses within a research model, it is necessary to analyze and report these indirect effects. Particularly in a parallel multiple mediator model, like in this study, it is essential to estimate specific indirect effects using phantom variables and review their significance. Based on his argument, the significance of indirect effects was confirmed through bootstrapping (ML, number of iterations 20,000, confidence interval 95%). The results verified the significance of the indirect effect of job satisfaction (job stress → job satisfaction → turnover intention, *b* = 0.159, *p* < 0.01) and organizational commitment (job stress → organizational commitment → turnover intention, *b* = 0.083, *p* < 0.01) in the relationship between job stress and turnover intention, as well as the parallel indirect effect of job satisfaction and organizational commitment (job stress → job satisfaction → organizational commitment → turnover intention, *b* = 0.125, *p* < 0.01).

## 5 Discussion

This paper aims to provide academic and practical implications for human resource management strategies suitable for youth sports facilities by elucidating the multifaceted relationships among job stress, job satisfaction, organizational commitment, and turnover intention. To achieve the set research objectives, Structural Equation Modeling (SEM) was utilized to test the research hypotheses regarding the relationships among these variables, and the following discussions were based on the results obtained.

Firstly, significant influences of job stress, job satisfaction, and organizational commitment on the turnover intention of Korean youth sports education facility instructors were observed (Hypotheses 3, 5, 6). The derived results suggest that job stress, job satisfaction, and organizational commitment can act as key psychological factors in mediating the intention to leave their current facilities and roles among Korean youth sports facility instructors. This finding aligns with previous research that reported job stress, job satisfaction, and organizational commitment as effective predictive variables for an individual’s turnover intention (Arshadi and Damiri, 2013; Chen and Kao, 2011; Cotton and Tuttle, 1986; Elci et al., 2012; Fasbender et al., 2019; Iqbal et al., 2014; Jehanzeb et al., 2013; Jou et al., 2013; Kuo et al., 2012; Romeo et al., 2020; Tett and Meyer, 1993; Yan et al., 2021a, 2021b).

Job stress, job satisfaction, and organizational commitment have long been known as one of the typical predictive variables for an individual’s turnover intention within an organization. In fact, previous studies on the mediation of turnover intention among various sports facility and sports education workers in Korea have reported that the level of turnover intention can vary depending on the levels of job stress (Jung, 2016; Lee, 2011; So et al., 2009), job satisfaction (Chon, 2007; Kim et al., 2012; Seo, 2018) and organizational commitment (Chung and Kang, 2014; Seo, 2012). Based on these insights, it is inferred that the significant influences of job stress, job satisfaction, and organizational commitment on the turnover intention of Korean youth sports education instructors have been elucidated. Particularly, previous research targeting early childhood and childcare teachers, whose job characteristics are similar to those of Korean youth sports education instructors (Choe, 2017; Ko and Sim, 2017; Lee and Lee, 2012; Lee and Pang, 2015; Oh and Chung, 2022), validates from a practical perspective that individual psychological factors like job stress, job satisfaction, and organizational commitment can play a role in mediating turnover intention.

Next, it was observed that job stress has a significant impact not only on the turnover intention of Korean youth sports education facility instructors (Hypotheses 1, 2) but also on their job satisfaction and organizational commitment. This result implies that the positive emotional responses of these instructors toward their job and organization, represented by job satisfaction and organizational commitment, can be diminished due to the negative emotions experienced during various job-related activities, represented by job stress. Furthermore, previous studies that have identified individual job stress in diverse work environments as a major predictive variable for job satisfaction and organizational commitment indirectly support the findings of this study (Beehr and Bhagat, 1985; Bhatti et al., 2016; Hayes et al., 2015; Iqbal et al., 2014; Jepson and Forrest, 2006; Tubre and Collins, 2000; Wang et al., 2020; Wu et al., 2021).

The effectiveness of job stress on job satisfaction and organizational commitment is likely due to the characteristic of job stress as a negative emotion. According to previous researchers, the stress experienced in the job process acts as a negative factor in the job and work environment of organizational members. In fact, studies targeting Korean sports instructors (Cho et al., 2002; Choi et al., 2013; Eun and Yeo, 2013; Lim et al., 2011) have highlighted that job stress acts as a negative factor in experiencing job satisfaction and organizational commitment.

Furthermore, it was observed that job satisfaction also has a significant impact on organizational commitment (Hypothesis 4). The derived result aligns with previous studies (Iqbal et al., 2014; Mowday et al., 1982; Oh et al., 2007; Williams and Hazer, 1986) that have identified job satisfaction as a precursor to organizational commitment. This suggests that the positive emotional response of Korean youth sports instructors toward their job can lead to an emotional sense of belonging to their sports education facility, namely organizational commitment.

On the other hand, based on the assertion by Bae (2017) that it is necessary to clarify indirect effects within a research model, even if no specific hypotheses have been set for them, the results of analyzing the significance of indirect effects within the model revealed significant indirect effects of organizational commitment in the relationship between job satisfaction and job stress, and also significant indirect effects of job stress in the relationship between job satisfaction, organizational commitment, and turnover intention.

The validity of the indirect effects, as derived, suggests that job stress, job satisfaction, organizational commitment, and turnover intention are interrelated in a complementary manner, with job stress acting as a key factor in determining the levels of job satisfaction, organizational commitment, and turnover intention. This implies not only the direct effectiveness of job stress on turnover intention but also its indirect effect through job satisfaction and organizational commitment. It indicates that the negative psychological and physiological responses experienced in job-related situations, namely job stress, play a significant psychological role in mediating the turnover intention of sports facility workers. In this context, researchers (Aghdasi et al., 2011; Arshadi and Damiri, 2013; Beehr and Bhagat, 1985; Chen and Kao, 2011; Elci et al., 2012; Iqbal et al., 2014; Jepson and Forrest, 2006; Jou et al., 2013; Kuo et al., 2012; Trivellas et al., 2013; Tubre and Collins, 2000; Wang et al., 2020; Wu et al., 2021) who have argued that job stress acts as a significant factor in individuals’ psychological and behavioral responses support the significant role of job stress as presented.

In summary, job stress, job satisfaction, and organizational commitment can be considered as effective human resource management strategies in preventing the turnover and attrition of employees from a psychological perspective. In this context, considering the complementary relationship that job stress, job satisfaction, and organizational commitment have with each other in influencing an individual’s intention to leave, and the direct and indirect effects they have on job satisfaction, organizational commitment, and turnover intention, job stress can be a crucial psychological factor in developing human resource management strategies suitable for the characteristics of workers in youth sports facilities. In other words, the stress response caused by excessive duties unique to the Korean youth sports education facility industry—such as educational guidance, vehicle operation, facility promotion, and emotional labor for parents (Schaufeli and Enzmann, 1998; Wang et al., 2020)—can serve as the most important human resource management strategy in the youth sports facility industry.

Therefore, based on the fact that managing competent human resources can significantly impact organizational competitiveness, especially in Korean youth sports education facilities where the importance of interaction between education service providers and consumers is emphasized (Jeon et al., 2023; Jung et al., 2023; Morrell et al., 2004; Seo, 2018), efforts should be made to secure excellent talents through human resource management strategies tailored to the characteristics of sports education instructors, from the perspective of mitigating job stress. Particularly, considering that many Korean youth sports education facilities operate on a relatively small scale as less than 5 workers, where facility operators can exert various influences directly on the employees, the operators should mediate the increasing turnover intention by understanding the major sources of stress for their instructors and undertaking empirical activities for mitigating, alleviating, and improving job stress. Through this, they should aim to enhance organizational competitiveness based on the acquisition of excellent human resources.

## 6 Conclusion

This paper is a study aimed at providing insights for developing effective human resource management strategies suitable for Korean youth sports education facilities, by elucidating the multifaceted relationships among job stress, job satisfaction, organizational commitment, and turnover intention. Through the research, it was academically suggested that the relationships among job stress, job satisfaction, organizational commitment, and turnover intention of Korean youth sports education facility workers could be structurally and complementarily manifested. Subsequently, it was proposed that facility operators should understand the main sources of stress for their instructors and undertake empirical activities for mitigating, alleviating, and improving job stress based on the significant role they play in managing individual job stress of sports instructors.

The implications presented by this study are academically and practically significant in providing a first step in related research, especially in a context where previous studies on the multifaceted verification of the causal relationships between job stress, job satisfaction, organizational commitment, and turnover intention among Korean youth sports education facility instructors are lacking. Therefore, additional efforts are needed to secure more objective and consistent evidence for the results obtained. In this context, the following suggestions are made as a conclusion.

Firstly, this study discusses the significance and role of job stress as a human resource management strategy in Korean youth sports education facilities through the clarification of the structural relationship among job stress, job satisfaction, organizational commitment, and turnover intention. This approach has limitations, as it only focused on these variables. Therefore, future research should provide diverse information on mitigating, alleviating, and improving job stress through the clarification of additional relationships with various variables.

Secondly, this study has limitations in clearly explaining the relationships among job stress, job satisfaction, organizational commitment, and turnover intention of workers in Korean youth sports facilities due to its cross-sectional research design. Furthermore, it also has limitations in not completely excluding the effects of exogenous variables such as salary, specific subsectors, and job characteristics. Hence, future related research should offer more useful information through deeper additional approaches such as longitudinal research designs or the introduction of control variables.

Third, the fact that the relationship between job stress, job satisfaction, organizational commitment, and turnover intention may vary depending on cultural and environmental characteristics cautions [e.g., Previous approaches mainly explain that job stress negatively affects organizational commitment, but Wang et al.’s (2020) study of Chinese university teachers also revealed that job stress can be positive for organizational commitment due to cultural characteristics] against theorizing and applying the observed findings to the youth physical education workforce from a universal perspective, and further research is needed to provide more meaningful information on this topic.

## Data Availability

The original contributions presented in the study are included in the article/supplementary material, further inquiries can be directed to the corresponding author.
